# Changes in the feeding ecology of an opportunistic predator inhabiting urban environments in response to COVID-19 lockdown

**DOI:** 10.1098/rsos.221639

**Published:** 2023-04-12

**Authors:** Marc Vez-Garzón, Joan Giménez, Antoni Sánchez-Márquez, Tomás Montalvo, Joan Navarro

**Affiliations:** ^1^ Institut de Ciències del Mar (ICM), CSIC, Passeig Marítim de la Barceloneta 37-49, 08003 Barcelona, Spain; ^2^ Servei de Vigilància i Control de Plagues Urbanes, Agencia de Salud Pública de Barcelona, Pl. Lesseps, 1, 08023 Barcelona, Spain

**Keywords:** anthropopause, COVID-19, feeding ecology, marine predator, urban wildlife, urban ecology

## Abstract

Urban-dwelling species present feeding and behavioural innovation that enable them to adjust to anthropogenic food subsidies available in cities. In 2020, the SARS-CoV-2 virus outbreak resulted in unprecedented reduction in the human activity worldwide associated with the human lockdown. This situation opened an excellent opportunity to investigate the capability of urban wildlife to cope with this anthropopause event. Here, we investigated the effects of the COVID-19 lockdown on the feeding strategies of the urban yellow-legged gull (*Larus michahellis*) population inhabiting the highly dense city of Barcelona (NE Spain). We compared the diet of chicks (through stomach content and stable isotope analyses) sampled randomly around the city of Barcelona before (2018 and 2019), during (2020) and after (2021) the COVID-19 lockdown. The results revealed that the anthropopause associated with the lockdown had an effect on the diet of this urban-dwelling predator. The diversity of prey consumed during the lockdown was lower, and consumption of urban birds (pigeons and parakeets) and marine prey (fishery discards and natural prey) decreased during the year of lockdown. Although it was not analysed, these diet changes probably were associated with variations in the availability of these resources due to the decrease in human activity during the lockdown. These results demonstrate the trophic flexibility of urban-dwelling species to cope with the changes in the availability of human-related anthropogenic resources in urban marine ecosystems.

## 1. Introduction

Human activity causes alterations in the functioning of natural ecosystems at global scale [1,2]. Among the different human impacts, the process of urbanization has notably affected biodiversity [3], displacing species from their natural habitats and reducing the size of their populations [4,5]. However, although human activity may negatively affect a large number of species, others are able to adjust and thrive in these urban environments [1]. Overall, urban-dwelling species tend to present high rates of feeding and behavioural innovation that enable them to exploit novel food resources present in these human-impacted habitats, which then could increase their survival and fitness [6,7]. This behavioural plasticity allows these species to adjust to new anthropogenic food resources present in the cities [7,8]. These flexible species are able to adjust their behaviours in human-altered landscapes successfully and can live in sympatry with humans in highly dense cities [6,9], sometimes providing ecosystem services, but very often disservices to society [10].

Among urban-dwelling wildlife, opportunistic gulls are clear examples of successful species that have become very common in urban areas around the world [11–14]. The high behavioural plasticity of these large seabirds has allowed them to exploit efficiently a great variety of novel trophic opportunities available in cities and surrounding habitats, including prey of human, marine, freshwater and terrestrial origin [13,15–18]. This is the example of the yellow-legged gull (*Larus michahellis*), a large-size gull with a widespread distributed along the Mediterranean region [19]. It is well adapted to urban life, efficiently preying on abundant urban birds such as rock pigeons (*Columba livia*) or monk parakeets (*Myiopsitta monachus*) and other resources associated with human activity such as human garbage and fishery discards [13,20–23]. Under scenarios of reduction of fishing activity or the closure of landfills or fisheries, natural populations of large gulls respond by changing their foraging strategies [24–26].

In 2020, the outbreak of the SARS-CoV-2 virus resulted in unprecedented reduction in human activity worldwide. In Spain, between March and May 2020, severe lockdown (COVID-19 lockdown) restrictions forced humans to confine themselves to their homes [27] and, similarly to other European countries, drastically reduced human activity to the essentials [28]. As a result, COVID-19 lockdown produced a reduction of human presence in the streets [29] and an alteration in the availability of human-sourced resources for opportunistic urban species [30,31]. This unique scenario offered an excellent opportunity to investigate the ability of urban wildlife to cope with this drastic anthropopause event [31]. Research in this area has revealed how the COVID-19 lockdown affected the behavioural patterns of urban mammals and birds associated with changes in food availability or human presence [32–35]. Overall, these studies evidenced how successful species inhabiting human-related habitats rapidly adjusted to the novel environmental conditions, directly related to their behavioural plasticity [33].

Here, we aimed to investigate the effects of the COVID-19 lockdown on the feeding behaviour of the urban yellow-legged gull population inhabiting the city of Barcelona (NE Spain), a highly populated European city severely affected by the COVID-19 anthropopause [29]. For this, we compared the diet (stomach content and stable isotope values) of chicks sampled randomly around the city of Barcelona before, during, and after the COVID-19 lockdown. Due to the high dependence on human resources of this species [13,36–38], we expected that the reduction in the human activity associated with the COVID-19 lockdown would affect the feeding behaviour of this urban gull population. For example, during the COVID-19 lockdown, the availability of fishery discards at sea, an important resource for this breeding colony [13,39], was reduced notably, associated with the reduction of the fishing activity in the waters close to Barcelona [40,41]. To compensate for the lack of fishing discards, we expected that urban yellow-legged gulls would increase the use of terrestrial habitats, increasing the consumption of resources such as urban birds (for example, rock pigeons), also an important part of their diet [13,21].

## 2. Methods

### 2.1. Fieldwork procedures

**Figure 1.**
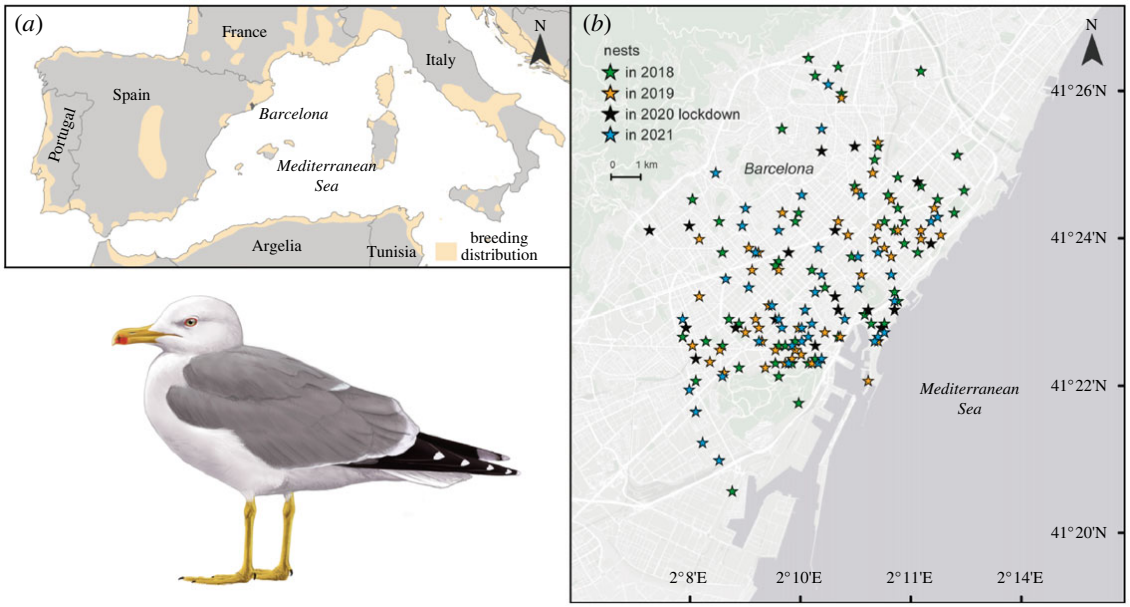
(*a*) General map showing breeding distribution of the yellow-legged gull (*Larus michahellis*) in Europe. (b) Distribution of the nests sampled in Barcelona during the pre-lockdown years (2018, 2019), the COVID-19 lockdown year (2020) and the year after (2021). The image of the yellow-legged gull was made by Martí Franch.

This study was developed in the city of Barcelona (NE Spain, figure 1) during the 2 years before the COVID-19 lockdown (2018 and 2019), the COVID-19 lockdown year (2020) and the year after (2021). Barcelona is a coastal urban area considered the second and eighth largest city of Spain and Europe regarding the number of habitants, respectively. The breeding population of yellow-legged gull of Barcelona has experienced a remarkable increment from one to five breeding pairs in the 1980s to around 300 pairs nowadays, distributed along the entire urban area [42,43]. Barcelona has different features that make it attractive to this opportunistic gull. The existence of elevated buildings provides rooftops that offer protection against predators or human disturbances. Furthermore, the urban environment of this city is surrounded by important fishing harbours, freshwater/river habitats, agricultural areas and waste management installations offerings a high variety of potential prey [13,20]. A significant activity is conducted in the fishing harbour of Barcelona and close coastal cities, with almost 3500 tons of fish caught every year [44]. This activity generates a large amount of fishing discards [45], being an important feeding resource for the yellow-legged gulls in this and other breeding areas [13,38,39]. In addition to marine prey, abundant urban birds such as rock pigeon and monk parakeet have been identified as key prey for this species [13,21].

To investigate the trophic ecology of yellow-legged gull, we analysed the stomach content and the stable isotope values of the new formed scapular feathers of 209 chicks collected and sacrificed along the city of Barcelona (only one chick per nest, and when we found more than one in a nest, we analysed the larger one; figure 1 for the distribution of nests) during the chick-rearing period (April–June). The Public Health Agency of Barcelona provided all these chicks. This is the responsible institution for the surveillance and control of wildlife species in the city (Legislative Decree 2/2008 of 15 April, DOGC; experimental permits 56L789 of Generalitat de Catalunya, Spain). To avoid potential differences in the diet between size/age [21], all chicks analysed showed similar tarsus length (ANOVA tests, *F_3,205_* = 2.24, *p* = 0.91; table 1) and body mass (*F_3,205_* = 0.72, *p* = 0.54; table 1). Moreover, all chicks were between one and three weeks of age, based on the principal component analysis (PCA) scores of the tarsus length and body mass, as these PCA scores increase linearly with the age of yellow-legged gull chicks [46]. We also discarded a potential effect on the diet in relation to the localization of the nests within the city, since unpublished GPS data of yellow-legged gull adults breeding in Barcelona during 2018, 2019 and 2021 showed similar foraging locations regardless of where the nest was placed within the city.

**Table 1.** Mean and s.d. of the body mass and tarsus length, and the stomach contents of yellow-legged gull chicks sampled in Barcelona before (2018, *n* = 82; 2019, *n* = 59), during the COVID lockdown (2020, *n* = 22) and the year after (2021, *n* = 46). %FO = frequency of occurrence of each prey in the stomachs. %N = contribution by number of each prey in the stomachs.

	2018		2019		2020 lockdown		2021	
body mass (g)	662.27 ± 207.54		666.54 ± 194.72		710.51 ± 129.46		707.13 ± 235.506	
tarsus length (cm)	62.83 ± 7.05		61.81 ± 5.28		66.16 ± 7.18		63.58 ± 6.94	
prey	%FO	%N	%FO	%N	%FO	%N	%FO	%N
**demersal marine prey**	31.71	22.75	42.37	43.56	27.27	44.44	26.09	25.00
*Boops boops*	19.51	11.38	27.12	32.67	27.27	44.44	17.39	11.36
*Phycis blennoides*	0	0	5.08	2.97	0	0	4.35	6.82
*Microchirus variegatus*	0	0	1.69	0.99	0	0	0	0
*Trisopterus capelanus*	2.44	1.20	3.39	1.98	0	0	0	0
*Nezumia aequalis*	3.66	1.80	0	0	0	0	0	0
*Pagellus acarne*	1.22	1.20	0	0	0	0	0	0
*Pagellus erythrinus*	1.22	0.60	0	0	0	0	0	0
*Micromesistius poutassou*	2.44	1.20	1.69	0.99	0	0	6.52	5.68
*Coelorinchus coelorinchus*	2.44	1.20	3.39	1.98	0	0	0	0
*Gaidropsarus biscayensis*	0	0	1.69	0.99	0	0	0	0
*Lesueurigobius sp*.	1.22	0.60	0	0	0	0	0	0
*Diplodus sp.*	2.44	1.20	0	0	0	0	0	0
*Trisopterus sp.*	2.44	1.20	0	0	0	0	0	0
Gadiformes	1.22	0.60	1.69	0.99	0	0	2.17	1.14
**pelagic marine prey**	8.54	5.99	18.64	12.87	0	0	4.35	4.55
*Gadiculus argenteus*	0	0	5.08	3.96	0	0	4.35	3.41
*Spicera smaris*	0	0	3.39	1.98	0	0	0	0
*Engraulis encrasicolus*	1.22	0.60	0	0	0	0	0	0
*Sardina pilchardus*	1.22	0.60	0	0	0	0	0	0
*Trachinotus ovatus*	1.22	0.60	0	0	0	0	0	0
*Trachurus mediterranius*	0	0	1.69	0.99	0	0	0	0
*Trachurus trachurus*	1.22	0.60	0	0	0	0	0	0
*Trachurus sp.*	6.10	4.19	0	0	0	0	2.17	1.14
*Spicera sp.*	0	0	3.39	1.98	0	0	0	0
Sparidae	0	0	6.78	3.96	0	0	0	0
**other marine prey**	28.05	13.77	6.78	3.96	9.09	11.11	21.74	12.50
cephalopods	6.10	2.99	0	0	4.55	5.56	4.35	3.41
marine crustaceans	4.88	2.40	0	0	0	0	2.17	1.14
unidentified fish	17.07	8.38	6.78	3.96	4.55	5.56	15.22	7.95
**urban birds**	67.07	34.73	61.02	35.64	27.27	33.33	76.09	40.91
*Columba livia*	19.51	9.58	40.68	23.76	13.64	16.67	36.96	20.45
*Myiopsitta monachus*	10.98	5.39	1.69	0.99	0	0	4.35	2.27
unidentified bird	40.25	19.76	20.34	10.89	13.64	16.67	34.78	18.18
**other terrestrial prey**	23.53	8.78	1.98	9.09	11.11	23.91	14.67	1.14
*Mus musculus*	1.22	0.60	0	0	0	0	0	0
*Rattus rattus*	1.22	0.60	0	0	0	0	0	0
unidentified mammal	28.05	13.77	3.39	1.98	9.09	11.11	23.91	12.50
invertebrate	7.32	4.19	0	0	0	0	2.17	1.14
**garbage**	7.32	3.59	3.39	1.98	0	0	6.52	3.41
*Gallus gallus domesticus*	6.10	2.99	1.69	0.99	0	0		
unidentified meat	0	0	1.69	0.99	0	0	2.17	1.14
unidentified vegetal	1.22	0.60	0	0	0	0	4.35	2.27
**inorganic items**	53.66	0	64.41	0	59.09	0	69.57	0
plastic items	47.56	0	55.93	0	54.55	0	56.52	0
metallic items	0	0	1.69	0	4.55	0	8.70	0
textile items	0	0	0	0	4.55	0	4.35	0
others	19.51	0	25.42	0	0	0	19.57	0

By combining the stomach content analysis with the determination of stable isotopes in feathers, we were able to benefit from the advantages and solve some of the biases associated with each methodology [38,47]. For example, stomach content analysis allows identifying the diet with a high taxonomic precision [48] but this methodology presents certain bias towards the overestimation of prey tissues/structures that are difficult to digest or towards preys ingested shortly before the sampling period [49]. The analysis of stable isotopes of *δ*^15^N (a proxy of trophic position; *δ*^15^N are positively related with the trophic position), *δ*^13^C (a proxy of the inshore, i.e. low *δ*^13^C values, and offshore habitats, i.e. high *δ*^13^C values) and *δ*^34^S (related to the terrestrial-marine origin of the prey; high *δ*^34^S values in terrestrial prey), is a complementary tool to infer the trophic niche of consumers during the period of the tissue analysed [50,51]. In our case, as the new scapular feathers are metabolically inert after synthesis, the feathers from yellow-legged chicks integrate the diet consumed and assimilated by the chicks during the feather growth (along two–three weeks) during the chick-rearing period [52].

### 2.2. Stomach content analysis

The stomach contents were identified and classified to the lowest possible taxonomic level, and counted to the lowest possible number of items. To determine the dietary importance of each prey type, for each year, we calculated two trophic metrics: %FO (frequency of occurrence of each prey in relation to the total number of stomachs analysed) and %N (contribution by number of each prey in relation to the total number of preys analysed). In addition to the trophic resources, we also calculated the %FO of plastic, textile fibres and metallic items. As a measure of trophic diversity, the Shannon's diversity index [53] was calculated for each year. Shannon's diversity index for the years 2018 (82 stomachs analysed), 2019 (59 stomachs analysed) and 2021 (46 stomachs analysed) was estimated by 1000 resamples of 22 random stomachs per year, 22 being the lowest number of stomachs analysed in the year 2020. Resampling procedure was done using the R package *mosaic* [54].

### 2.3. Stable isotope analysis

Newly formed scapular feathers were cleaned, dried and the entire feathers were powdered and between 0.28 and 0.33 mg for *δ*^13^C and *δ*^15^N analysis and around 1.5 mg for *δ*^34^S analysis were packed into tin capsules and sent to the Stable Isotopes Lab of the *Estación Biológica de Doñana* (EBD-CSIC; www.ebd.csic.es/lie/index.html), where stable isotopic analyses were performed. The samples were combusted at 1020°C using a continuous flow isotope ratio mass spectrometry system (Thermo Electron) by means of a Flash HT Plus elemental analyser interfaced with a Delta V Advantage mass spectrometer which applies international standards. Stable isotope ratios were expressed in the standard *δ*-notation (‰) relative to troilite from the Canyon Diablo Meteorite (*δ*^34^S), atmospheric N_2_ (*δ*^15^N) and Vienna Pee Dee Belemnite (*δ*^13^C). The measurement error (± s.d.) was ±0.1, ±0.1 and ±0.2*‰* for *δ*^34^S, *δ*^13^C and *δ*^15^N, respectively.

*δ*^13^C, *δ*^15^N and *δ*^34^S values from feathers were used to infer the isotopic niche volume (N_R_) of the yellow-legged gulls for each sampling year. The N_R_ is defined as a three-dimensional volume contained in a multi-variate space that contains the probability of finding a specific individual from a certain year in 40% of the core isotopic niche volume of that specific year. We used 40% of the total niche volume because it is a percentage commonly associated with the core N_R_ of a species [55], and in this case, the core N_R_ of the gulls for each year was calculated using a Bayesian probabilistic method implemented in the R package nicheROVER [56]. N_R_ estimation was based on 1000 random projections generated by the Bayesian probabilistic method, and they were used to calculate the mean N_R_ and overlap between years. Ten of these random projections of the bivariate niches were used for plotting in order to ease the visualization. As N_R_ was estimated with Bayesian statistics, we calculated the probability that the posterior distribution of 2020 is smaller than the rest of the years.

### 2.4. Statistical analysis

We applied PERMANOVA and pairwise tests based on a Bray–Curtis distance matrix and square-root transformed data to compare the %N, %FO, *δ*^15^N, *δ*^13^C and *δ*^34^S values between the years before (2018 and 2019), the COVID-19 lockdown year (2020) and the year after (2021). The method calculates a pseudo-*F* statistic, analogue to the traditional *F* statistic of ANOVA tests, using permutation procedures to obtain the *p*-values [57]. In the case of differences among years, a similarity percentage analysis (SIMPER tests) with 999 permutations was performed [57] to identify which prey type contributed most to the observed diet differences among years. PERMANOVA and SIMPER tests were conducted with PRIMER-E v. 6 software [57].

## 3. Results

### 3.1. Stomach content

**Figure 2.**
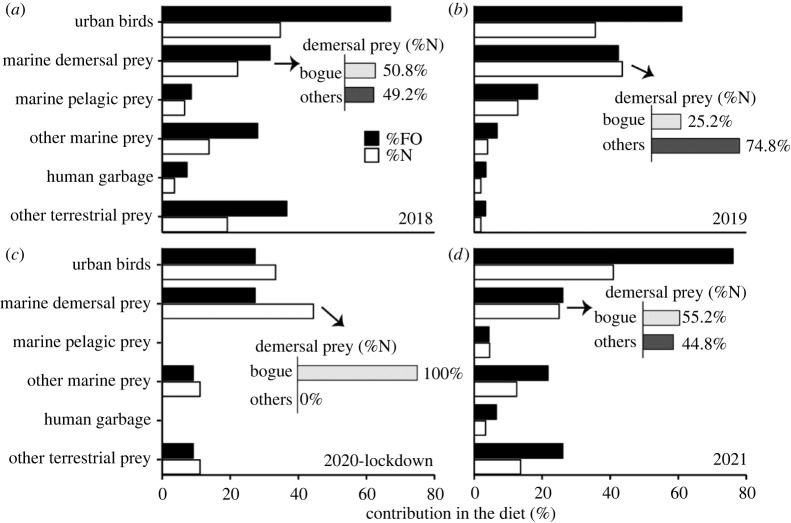
Prey categories found in the stomach content of yellow-legged gull chicks sampled in Barcelona before ((*a*) 2018: *n* = 82 chicks; (*b*) 2019: *n* = 59 chicks), during the COVID lockdown ((*c*) 2020: *n* = 22 chicks) and the year after ((*d*) 2021: *n* = 46) calculated as the percentage of stomachs (%FO) and the percentage of preys (%N) for each category. The (%N) of the bogue (*Boops boops*) in relation to the other demersal fish prey is also indicated.

**Figure 3.**
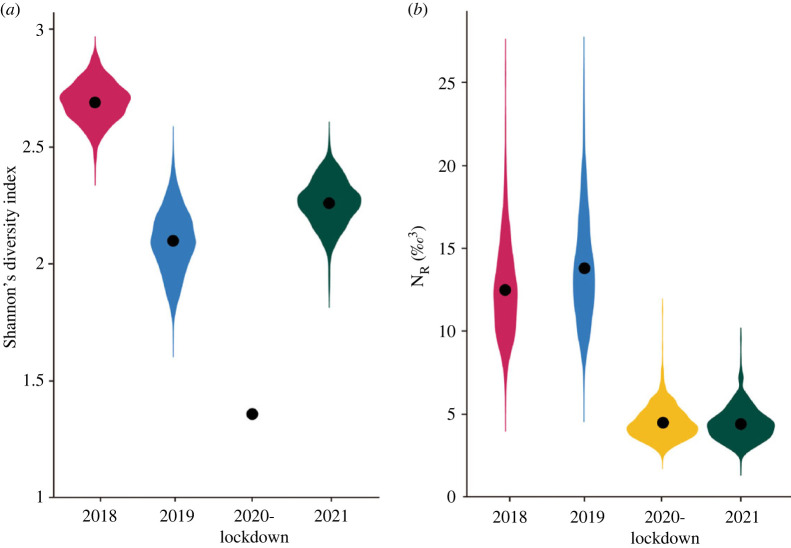
(*a*) Shannon index values based on the stomach contents and (*b*) trophic niche volume (N_R_ ‰^3^) inferred from *δ*^13^C, *δ*^15^N and *δ*^34^S isotopic values of newly formed scapular feathers of yellow-legged gull chicks sampled in Barcelona before (2018 and 2019), during the COVID lockdown (2020) and the year after (2021). For the years 2018, 2019 and 2021, the Shannon index was estimated from 1000 resamples of 22 random stomachs (the number of stomachs analysed in 2020).

Overall for all years, the stomach content of yellow-legged gull chicks was mainly composed of urban birds, followed by marine demersal prey, with less importance of marine pelagic prey, other marine prey, other terrestrial prey and human garbage (table 1, figure 2). However, we found differences among years in prey found in the stomachs (%N; pseudo-*F*_3,207_ = 3.27, *p* = 0.006; %FO; pseudo-*F*_3,207_ = 1.02, *p* = 0.02; table 1, figures 2 and 3) and in the Shannon diversity index (figure 3). In particular, during the COVID-19 lockdown year, urban birds such as pigeons (*Columba livia*) or monk parakeets (*Myiopsitta monachus*) decreased in both %N and %FO in comparison with the previous years (2018 and 2019) and the year after the lockdown (2021) (SIMPER tests comparing %N and %FO between 2020 and the other years always present *p* < 0.05; table 1, figure 2). Marine pelagic prey, although overall were not an important part of the yellow-legged gull diet, during the COVID-19 lockdown, this group was not detected in any of the 22 stomachs analysed this year (table 1, figure 2). In relation to the other groups, a decrease in the %N of marine demersal prey between the 2019 and the COVID-19 lockdown year (SIMPER tests, *p* = 0.01) was found (table 1, figure 2). Within marine demersal prey, the predominant demersal species during all years was the bogue (*Boops boops*), but it was the only marine prey found in the stomach content of the yellow-legged gull chicks during the COVID-19 lockdown year (table 1, figure 2). Other items associated with anthropogenic origin such as plastic remains were also found in the stomachs, with elevated %FO values that ranged between 53% and 69%, but without differences among years (table 1).

Regarding the Shannon diversity index, the COVID-19 lockdown year showed the lowest values (1.52) whereas higher diversity values were found in 2018 (mean ± s.d. = 2.23 ± 0.36), followed by 2021 (2.01 ± 0.34) and 2019 (1.79 ± 0.45) (figure 3*a*).

### 3.2. Stable isotopes

We found significant differences among years for the three stable isotopes analysed (*δ*^15^N, pseudo-*F*_3,115_ = 2.14, *p* < 0.0001; *δ*^13^C, pseudo-*F*_3,115_ = 5.63, *p* < 0.0001; *δ*^34^S, pseudo-*F*_3,115_ = 1.94, *p* < 0.0001; table 2, electronic supplementary material, table S1). Specifically, SIMPER tests indicated that during the COVID-19 lockdown year, yellow-legged gull chicks showed higher *δ*^15^N and *δ*^13^C values than during the 2 previous years and the year after (*p* < 0.05; table 2). In the case of the *δ*^34^S values, yellow-legged gull chicks showed significantly higher values during 2018 in comparison with the other years (*p* < 0.05; table 2).

**Table 2.** Number of samples (*n*), mean and s.d. of *δ*^13^C, *δ*^15^N, *δ*^34^S and the niche volume (N_R_) values of newly formed scapular feathers of yellow-legged gull chicks sampled in Barcelona before (2018 and 2019), during the COVID lockdown (2020) and the year after (2021). See electronic supplementary material, table S1 for more information.

year	*n*	*δ*^15^N (‰)	*δ*^13^C (‰)	*δ*^34^S (‰)	N_R_ (‰^3^)
2018	29	9.52 ± 0.88_a_	−20.35 ± 0.89_a_	13.72 ± 2.67_a_	12.48 ± 2.99
2019	29	9.68 ± 0.71_a_	−20.82 ± 1.09_b_	11.35 ± 2.88_b_	14.01 ± 3.14
2020 lockdown	32	10.16 ± 0.61_b_	−19.57 ± 0.57_c_	12.23 ± 2.09_b_	4.43 ± 0.99
2021	29	9.43 ± 0.72_a_	−20.21 ± 0.87_a_	10.71 ± 2.94_b_	4.37 ± 1.00

Values with the same subscript indicate no significant differences between years based on SIMPER tests.

**Figure 4.**
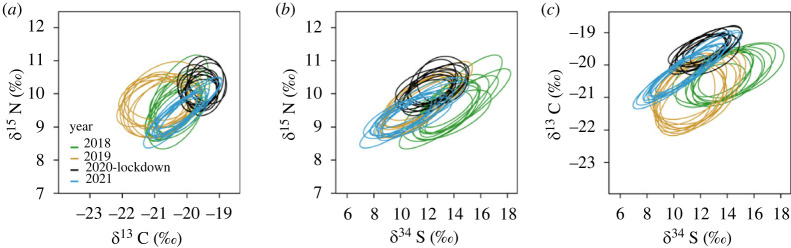
Two-dimensional isotopic niche based on ellipse areas (‰^2^) from the (*a*) *δ*^15^N and *δ*^13^C values, (*b*) *δ*^15^N and *δ*^34^S values and (*c*) *δ*^13^C and *δ*^34^S values of newly formed scapular feathers of yellow-legged gull chicks sampled in the city of Barcelona before (2018: *n* = 29 chicks; 2019: *n* = 29 chicks), during the COVID lockdown (2020: *n* = 32 chicks) and the year after (2021: *n* = 21 chicks).

The isotopic niche volume (N_R_) showed higher values during the 2 years before the year of the COVID-19 lockdown and the year after (figure 3*b*, table 2). Specifically, we found that 2020 had a greater than 99% probability of being smaller than 2018 and 2019, and a 47.8% probability of being smaller than 2021 (figure 3*b*). In addition, N_R_ volume showed a partial segregation between the COVID-19 lockdown and the years before and after (table 3, figure 4).

**Table 3.** N_R_ overlap of yellow-legged gull chicks sampled in Barcelona before (2018 and 2019), during the COVID lockdown (2020) and the year after (2021). As the volume is different depending on the year, the overlap is asymmetric (i.e. if you compare for example 2018 with the volume of 2019 or 2019 with the volume of 2018).

overlap (%)	2018	2019	2020 lockdown	2021
2018		16.8 [7.0–31.5]	3.3 [0.8–7.1]	5.6 [2.0–10.8]
2019	13.6 [4.7–26.7]		6.3 [2.7–11.7]	9.6 [5.2–15.3]
2020 lockdown	5.9 [0.4–20.8]	20.1 [3.9–46.7]		16.4 [5.9–30.7]
2021	16.9 [3.8–38.0]	36.5 [15.3–60.8]	18.5 [9.2–30.3]	

## 4. Discussion

The present study revealed how the interruption of human activity associated with the strict COVID-19 lockdown in Barcelona, which overlapped with the chick-rearing period of the urban yellow-legged gulls, forced a change in their feeding behaviour. During the COVID-19 lockdown year, the diet of yellow-legged gull chicks showed smaller trophic niche and prey diversity, less importance of urban birds, marine pelagic prey and human garbage, and an increase of marine demersal prey in their diet, and an enrichment in *δ*^13^C and *δ*^15^N values in their feathers in comparison with the previous and the subsequent years. These results provided evidence of how this opportunistic predator is able to adjust and modify its diet to cope with the constraint associated with a drastic change in the availability of different resources mainly associated with humans in an urban marine ecosystem.

During the COVID-19 lockdown, the fishing activity along the northwestern Mediterranean Sea, an important feeding ground for yellow-legged gulls inhabiting Barcelona [13,39], was reduced considerably [40,41]. This situation affected the abundance and availability of fishery discards provided by trawlers and purse-seiners [40], important human-related resources for opportunistic seabirds [13,46,58]. For this reason, we expected a clear reduction in the presence of marine resources in the diet of the yellow-legged gull chicks during the COVID-19 lockdown year. This was evident in the absence of marine pelagic fish in the stomachs of the chicks during the lockdown year, reflecting the low availability due to the reduction of purse-seine activity, a fishing activity that provides a high amount of pelagic fish via discards for Mediterranean seabirds [59,60]. However, although a similar pattern was expected for marine demersal prey, only available for surface feeder seabirds through trawling discards [61,62], here we found that during the lockdown, demersal prey group was an important food item for yellow-legged gulls. Specifically, the bogue, the main demersal prey identified during all the years analysed and considered one of the most discarded species by the trawling fleet in the western Mediterranean [45], was the only demersal prey identified in the stomachs during the lockdown period. Although bogue is present in Mediterranean deep waters up to 350 m, it is a species that can also be found close to the sea surface in shallow waters [63], where gulls are able to capture them. Moreover, it is possible that the presence of the bogue in shallow waters increased during the lockdown associated with the reduction in human mobility [64,65], increasing the availability of this prey in particular habitats such as beaches or harbours, commonly visited by urban yellow-legged gulls [13]. Both hypotheses could explain why the bogue is the main demersal prey in the diet of yellow-legged gull and it was the only demersal fish present in the stomachs during the COVID-19 lockdown year. Moreover, we could interpret this result as the necessity of adults to provide fish of better nutritional quality than terrestrial resources to the chicks to compensate for the lack of other marine resources often available in other years.

Isotopically, the enriched *δ*^13^C and *δ*^15^N values are related to diets with a high proportion of demersal and/or nearshore species [66–68]. For this, the enriched *δ*^13^C and *δ*^15^N values during the lockdown reflected the absence of marine pelagic prey and the relatively high consumption of bogue showed in the stomach contents. Alternatively, the increase of *δ*^15^N values during the COVID-19 lockdown year could be reflecting the use of their protein stores by the chicks during the feather formation [52] associated with a potential reduction in the quantity of food to the chicks provided by their parents due to the reduction of easy-to-catch resources such fishery discards [39].

To compensate for the lack of fishing discards during the lockdown [40], we expected that urban yellow-legged gulls increased the consumption of alternative resources such as urban birds, considered one of the main resources consumed by urban yellow-legged gulls in Barcelona [13,21]. The reason for this initial prediction was that urban birds such as pigeons or invasive monk parakeets might have become more accessible to be captured by the gulls during the lockdown due to the reduction of human and vehicle presence in Barcelona [27,28]. However, based on our results, urban birds were consumed in a lower proportion during the lockdown than during the other years. In fact, it is important to remark that during the lockdown, the proportion of urban birds found in the stomachs was lower than the proportion of marine resources. Thus, why did urban bird consumption not increase during the COVID-19 lockdown year? Two complementary explanations directly related to the availability of these prey for the urban yellow-legged gulls could explain this behaviour. The reduction in the number of vehicles associated with the lockdown [27,28] probably reduced the number of dead urban birds associated with vehicle collisions [69,70], reducing the availability of bird carcases for urban gulls [15]. In addition to the scavenging behaviour, the yellow-legged gull also preys on rock pigeons when they are concentrated in large groups of hundreds of individuals feeding on food provided by citizens [71,72]. The lack of human food provisioning in urban habitats during the lockdown probably caused a reduction in the presence of these large aggregations of rock pigeons, dispersing them throughout the city [30]. Thus, this change in the aggregation patterns of urban birds during the lockdown probably reduced the success of capture by yellow-legged gulls [73]. However, the similar *δ*^34^S values between the lockdown and the year before and after, suggest that despite the low consumption of urban birds, their diet assimilation did not change [51,74]. It is also important to mention that the predation of pigeons and parakeets explains the presence of some pathogens [75] or seeds of plants [76] carried by gulls that potentially could be spread to humans [77–79] or to natural habitats [21], respectively. For this, indirectly, the low consumption of urban birds in the diet of yellow-legged gull during the lockdown could reduce the potential spread of both pathogens and seeds during 2020.

Regarding the diet diversity metrics, we found some differences between the Shannon diversity index estimated with the stomach content and the isotopic niche (N_R_). During the COVID-19 lockdown year, the Shannon index using the stomach content information showed a lower value, clearly reflecting that during this year only one species of demersal and pelagic marine prey was detected in the stomachs. By contrast, N_R_ metric indicated that in both the lockdown and the following year, the yellow-legged chicks showed smaller trophic niche volumes, a clear reflection that the diet was less diverse than during the years prior to lockdown [80].

In conclusion, as we predicted, the anthropopause associated with the COVID-19 lockdown apparently had an effect on the feeding ecology of this urban-dwelling predator. The diversity of prey consumed during the lockdown was lower than in the years before and after. In addition, the predation on urban birds and marine prey decreased during the year of lockdown, associated with changes in the availability of these resources due to a drastic decrease in human activity and mobility. In addition to the observed change in stomach contents, stable isotope values in yellow-legged gulls' feathers also reflects the feeding changes produced by the anthropopause. The results of this study, therefore, demonstrate the trophic flexibility of this species to cope with the changes in the availability of human-related anthropogenic resources in urban marine ecosystems.

## Data Availability

The raw stable isotope data is provided in the electronic supplementary material. The data are provided in the electronic supplementary material [81].
